# Supplementary material for: The Development of a Personalized Symptom Management Mobile Health Application for Persons Living with HIV in China
Source: J Pers Med. 2021 Apr 25;11(5):346. doi: 10.3390/jpm11050346 (PMC8146530; doi:10.3390/jpm11050346)
Supplement: Supplementary file 1 [file jpm-11-00346-s001.zip › jpm-1181058-supplementary.pdf]

Supplementary material 1

Table S1 Symptom management app content source

Organization/Author	Title/Website	Type
Shanghai Public Health Center Affiliated to Fudan University & Fudan University Evidence-based Nursing Center	AIDS clinical nursing practice guideline [1]	Guideline
AIDS Professional Group, Society of Infectious Diseases, Chinese Medical Association	Third edition of the guidelines for diagnosis and treatment of HIV/AIDS (2015) [2]	Guideline
The Association of Nurses in AIDS Care	Developing evidence-based symptom management guidelines in HIV care: what have we learned? [3]	Guideline
	Managing fever and febrile symptoms in HIV: evidence-based approaches [4]	Guideline
	Evidence-based clinical practice guidelines for managing depression in persons living with HIV [5]	Guideline
	Anxiety symptoms in HIV-infected individuals [6]	Guideline
	Evidence-based pain guidelines in HIV care [7]	Guideline
	Assessing and treating forgetfulness and cognitive problems in adults with HIV [8]	Guideline
	HIV-associated wasting [9]	Guideline
	Fatigue in HIV and AIDS: an analysis of evidence [10]	Guideline
	Sleep disturbances in persons living with HIV [11]	Guideline
	ANAC's core curriculum for HIV/AIDS nursing (Third edition) [12]	Book
European AIDS Clinical Society Department of Health and Human Services	EACS guidelines [13]	Guideline

U.S. Department of Health and Human Services	Guidelines for the use of antiretroviral agents in HIV-1-infected adults and adolescents (Panel on antiretroviral guidelines for adults and adolescents) [14]	Guideline
	Guidelines for the use of antiretroviral agents in pediatric HIV infection (Panel on antiretroviral therapy and medical management of HIV - infected children) [15]	Guideline
	Guidelines for prevention and treatment of opportunistic infections in HIV-infected adults and adolescents (Panel on opportunistic infections in HIV-infected adults and adolescents) [16]	Guideline
	Physical activity guidelines [27]	Guideline
International Association of Providers of AIDS Care	Nutrition guidelines for people with HIV [17]	Guideline
British HIV Association	BHIVA guidelines for the routine investigation and monitoring of adult HIV-1-positive individuals 2016 [18]	Guideline
	BHIVA guidelines for the treatment of HIV-1-positive adults with antiretroviral therapy 2015 (2016 interim update) [19]	Guideline
British Columbia Center for Excellence in HIV - AIDS	Primary care guidelines for the management of HIV/AIDS in British Columbia [20]	Guideline
New York State Department of Health AIDS Institute	Depression and mania guideline [21]	Guideline
	Anxiety disorders guidelines [22]	Guideline
	Insomnia screening and treatment [23]	Guideline
	Cognitive disorders guideline [24]	Guideline
	Somatic symptom guideline [25]	Guideline
	Oral health complications [26]	Guideline

The Office of AIDS Research Advisory Council (OARAC)	Recommendations for use of antiretroviral drugs in pregnant HIV-1-infected women for maternal health and interventions to reduce perinatal HIV transmission in the United States [28]	Guideline
Hongzhou Lu, Yan Hu	Practical AIDS care [29]	Book
Hongzhou Lu	Clinical pathway of AIDS and related diseases (Second Edition) [30]	Book
Anqiu Fu	Psychological counselor training course (Second Edition) [31]	Book
University of California, San Francisco	Symptom management strategies: A manual for people living with HIV/AIDS [32]	Manual
Health Resources and Services Administration HIV/AIDS Bureau	Guide for HIV/AIDS clinical care [33]	Guide
Canadian AIDS Treatment Information Exchange (CATIE)	A practical guide to nutrition for people living with HIV [34]	Guide
	A practical guide to herbal therapies for people living with HIV [35]	Guide
	A practical guide to HIV drug side effects for people living with HIV [36]	Guide
	A practical guide to a health body for people living with HIV [37]	Guide
	Managing your health: a guide for people living with HIV [38]	Guide
	HIV and emotional wellness [39]	Manual
HIV iBASE	HIV and quality of life: guide to fewer side effects, better health and longer life [40]	Manual
World Health Organization	https://www.who.int/	
UNAIDS	https://www.unaids.org/en	
National Health Commission of the People's Republic of China	http://www.nhc.gov.cn/	
Chinese Center for Disease Control and Prevention	http://www.chinacdc.cn/	

China HIV/AIDS Information Network	http://www.chain.net.cn/
Shanghai Municipal Center for Disease Control & Prevention	http://www.scdc.sh.cn/
Shanghai Public Health Center Affiliated to Fudan University	http://www.shaphc.org/
Fudan University Evidence-based Nursing Center	http://nursing.ebn.fudan.edu.cn/
Chinese Nutrition Society	https://www.cnsoc.org/

References:

1. Fu L. *Development of the HIV/AIDS Clinical Nursing Practice Guidelines;* Fudan Univerity: Shanghai, China, 2014.
2. AIDS Professional Group, Society of Infectious Disease, Chinese Medical Association. Third Edition of the Guidelines for Diagnosis and Treatment of HIV/AIDS (2015). *Chin. J. Intern. Med.* **2015**, 8, 385–401, doi:10.3760/cma.j.issn.1674-2397.2015.05.001.
3. Voss, J.G. Developing Evidence-Based Symptom Management Guidelines in HIV Care: What Have We Learned? *J. Assoc Nurses AIDS Care*. **2013**, 24, S1-4, doi:10.1016/j.jana.2012.10.001.
4. Holtzclaw, B.J. Managing Fever and Febrile Symptoms in HIV: Evidence-Based Approaches. *J. Assoc Nurses AIDS Care*. **2013**, 24, S86-102, doi:10.1016/j.jana.2012.07.002.
5. Relf, M.V.; Eisbach, S.; Okine, K.N.; Ward, T. Evidence-Based Clinical Practice Guidelines for Managing Depression in Persons Living with HIV. *J. Assoc Nurses AIDS Care*. **2013**, 24, S15-28, doi:10.1016/j.jana.2012.05.007.
6. Kemppainen, J.K.; MacKain, S.; Reyes, D. Anxiety Symptoms in HIV-Infected Individuals. *J. Assoc Nurses AIDS Care.* **2013**, *24*, S29-39, doi:10.1016/j.jana.2012.08.011.

7. Newshan, G.; Staats, J.A. Evidence-Based Pain Guidelines in HIV Care. *J. Assoc Nurses AIDS Care.* **2013**, *24*, S112-126, doi:10.1016/j.jana.2012.05.006.

8. Vance, D.E.; Fazeli, P.L.; Moneyham, L.; Keltner, N.L.; Raper, J.L. Assessing and Treating Forgetfulness and Cognitive Problems in Adults with HIV. *J. Assoc Nurses AIDS Care.* **2013**, *24*, S40-60, doi:10.1016/j.jana.2012.03.006.

9. Keithley, J.K.; Swanson, B. HIV-Associated Wasting. *J. Assoc Nurses AIDS Care.* **2013**, *24*, S103-111, doi:10.1016/j.jana.2012.06.013.

10. Barroso, J.; Voss, J.G. Fatigue in HIV and AIDS: An Analysis of Evidence. *J. Assoc Nurses AIDS Care.* **2013**, *24*, S5-14, doi:10.1016/j.jana.2012.07.003.

11. Taibi, D.M. Sleep Disturbances in Persons Living with HIV. *J. Assoc Nurses AIDS Care.* **2013**, *24*, S72-85, doi:10.1016/j.jana.2012.10.006.

12. Association of Nurses in AIDS Care *ANAC's Core Curriculum for HIV/AIDS Nursing (Third Edition)*; Jones & Bartlett Learning: Burlington, 2009.

13. European AIDS Clinical Society. EACS guidelines Version 8.2. 2017. Available online: https://www.eacsociety.org/files/guidelines_8.2-english.pdf (accessed on January 2nd, 2017).

14. U.S. Department of Health and Human Services. Guidelines for the use of antiretroviral agents in HIV-1-infected adults and adolescents. 2016. Available online: https://aidsinfo.nih.gov/guidelines/ (accessed on July 30th, 2016).

15. U.S. Department of Health and Human Services. Guidelines for the use of antiretroviral agents in pediatric HIV infection. 2016. Available online: https://aidsinfo.nih.gov/guidelines/ (accessed on July 30th, 2016).

16. U.S. Department of Health and Human Services. Guidelines for prevention and treatment of opportunistic infections in HIV - infected adults and adolescents. 2016. Available online: https://aidsinfo.nih.gov/guidelines/ (accessed on July 30th, 2016).

17. International Association of Providers of AIDS Care. Nutrition guidelines for people with HIV. 2014. Available online: http://aidsinfonet.org/fact_sheets/view/800 (accessed on August 28th, 2014).

18. British HIV Association. BHIVA guidelines for the routine investigation and monitoring of adult HIV-1-positive individuals 2016. 2016.Available online: https://www.bhiva.org/monitoring-guidelines (accessed on December 31st, 2016).

19. British HIV Association. BHIVA guidelines for the treatment of HIV-1-positive adults with antiretroviral therapy 2015 (2016 interim update). 2016. Available online: https://www.bhiva.org/HIV-1-treatment-guidelines (accessed on December 31st, 2016).

20. British Columbia Center for, Excellence in HIV-AIDS. Primary care guidelines for the management of HIV/AIDS in British Columbia. 2015. Available online: http://www.cfenet.ubc.ca/sites/default/files/uploads/primary-care-guidelines/primary-care-guidelines_015-09-15.pdf (accessed on August 31st, 2015).

21. New York State Department of Health AIDS Institute. Depression and mania guideline. 2010. Available online: http://www.hivguidelines.org/mental-health-in-hiv/depression-mania-in-hiv/ - tab_0 (accessed on December 31st, 2010).

22. New York State Department of Health AIDS Institute. Anxiety disorders guidelines. 2006. Available online: http://www.hivguidelines.org/mental-health-in-hiv/ (accessed December 31st, 2006).

23. New York State Department of Health AIDS Institute. Insomnia screening and treatment (Quick reference guide). 2013. Available online: https://www.hivguidelines.org/mental-health-in-hiv/somatic-symptoms-in-hiv/ - tab_4 (accessed on December 31st, 2013).

24. New York State Department of Health AIDS Institute. Cognitive disorders guideline. 2007. Available online: https://www.hivguidelines.org/mental-health-in-hiv/cognitive-disorders-in-hiv/ - tab_0 (accessed on December 31st, 2007).

25. New York State Department of Health AIDS Institute. Somatic symptom guideline. 2008. Available online: https://www.hivguidelines.org/mental-health-in-hiv/somatic-symptoms-in-hiv/ - tab_4 (accessed on December 31st, 2008).

26. New York State Department of Health AIDS Institute. Oral health complications. 2008. Available online: https://www.hivguidelines.org/hiv-care/oral-health/ (accessed on December 31st, 2008).

27. U.S. Department of Health and Human Services. 2008 Pyhsical activity guidelines for americans. 2008. Available online: https://health.gov/sites/default/files/2019-09/paguide.pdf (accessed on December 31st, 2008).

28. The Office of AIDS Research Advisory Council. Recommendations for use of antiretroviral drugs in pregnant HIV-1-infected women for maternal health and interventions to reduce perinatal HIV transmission in the United States. 2016. Available online: https://aidsinfo.nih.gov/contentfiles/lvguidelines/perinatalgl.pdf (accessed on December 31st, 2016).

29. Lu, H.; Hu, Y. *Practical AIDS Care*; Shanghai Science & Technical Publishers: Shanghai, 2014.

30. Lu, H. *Clinical Pathway of AIDS and Related Diseases (Second Edition)*; Shanghai Science & Technical Publishers: Shanghai, 2015.

31. Fu, A. *Psychological Counselor Training Course (Second Edition)*; East China Normal University Press: Shanghai, 2017.

32. San Francisco Regents *Symptom Management Strategies: A Manual for People Living with HIV/AIDS*; University of California, San Francisco Regents, School of Nursing: San Francisco, CA, 2004.

33. Health R. Guide for HIV/AIDS clinical care. 2014. Available online: https://aidsetc.org/guide/contents (accessed on December 31st, 2014).

34. Canadian AIDS Treatment Information Exchange, Canada's Source for HIV and Hepatitis C Information. A practical guide to nutrition for people living with HIV. 2007. Available online: https://www.catie.ca/en/practical-guides/nutrition (accessed on December 31st, 2007).

35. Canadian AIDS Treatment Information Exchange, Canada's Source for HIV and Hepatitis C Information. A practical guide to herbal therapies for people living with HIV. 2005. Available online: https://www.catie.ca/ga-pdf.php?file=pdf/PG_Herb/HERBAL_guide_english_2005.pdf (accessed on July December 31st, 2005).

36. Canadian AIDS Treatment Information Exchange, Canada's Source for HIV and Hepatitis C Information. A practical guide to HIV drug side effects. 2013. https://www.catie.ca/en/practical-guides/hiv-drug-side-effects (accessed on December 31st, 2013).

37. Canadian AIDS Treatment Information Exchange, Canada's Source for HIV and Hepatitis C Information. A practical guide to a health body for people living with HIV. 2015. Available online: https://www.catie.ca/en/practical-guides/healthy-body (accessed on December 31st, 2015).

38. Canadian AIDS Treatment Information Exchange, Canada's Source for HIV and Hepatitis C Information. Managing your health: a guide for people living with HIV. 2014. Available online: https://www.catie.ca/en/practical-guides/managing-your-health (accessed on December 31st, 2014).

39. Canadian AIDS Treatment Information Exchange, Canada's Source for HIV and Hepatitis C Information. HIV and emotional wellness. 2013. Available online: http://www.catie.ca/en/practical-guides/emotional-wellness (accessed on December 31st, 2013).

40. HIV i-BASE. HIV and Quality of life: guide to fewer side effects, better health and longer life. 2016. Available online: http://i-base.info/guides/side/pdf (accessed on August 1st, 2016).

Supplementary material 2

Table S2 Scores in terms of evidence-based, situational suitability, practicability, cost-effectiveness, and understandability in the group discussion

	Experts (n = 10)																			
	Sufficient evidence				Situational suitability				Practicability				Cost-effectiveness				Understandability			
	1	2	3	4	1	2	3	4	1	2	3	4	1	2	3	4	1	2	3	4
**Health tracking**																				
Blood test	0	0	5	5	0	0	1	9												
Prescription	0	0	2	8	0	0	3	7												
Medication adherence	0	0	1	9	0	1	3	6												
Symptoms tracking	0	0	3	7	0	1	3	6												
**Self-assessment**																				
Symptom assessment	0	0	3	7	0	4	1	5												
Medication adherence	0	1	2	7	0	1	2	7												
Quality of life	0	0	3	7	0	0	3	7												
Lifestyle	0	0	3	7	0	3	3	4												
Stigma experience	0	1	3	6	0	7	3	0												
Depression	0	0	0	10	0	4	3	3												
Anxiety	0	0	0	10	0	4	1	5												
Sleep diary	0	0	0	10	0	4	5	1												
Emotion diary	0	2	3	5	0	1	2	7												
Insomnia	0	0	3	7	0	4	4	2												
**Fatigue**																				
Change cognition	0	0	2	8	0	0	3	7	0	0	3	7	0	0	2	8	0	0	1	9

Ask for help	0	0	0	10	0	0	1	5	0	1	1	8	0	0	0	10	0	0	1	9
Exercise	0	0	3	7	0	0	4	6	0	0	4	6	0	0	1	9	0	0	1	9
Change diet	0	0	2	8	0	0	3	7	0	0	3	7	0	0	2	8	0	0	1	9
Complementary therapy	1	2	4	3	0	0	5	5	0	2	4	4	0	1	4	5	0	1	3	6
**Depression**																				
Change cognition	0	0	1	9	0	0	1	9	0	0	3	7	0	0	0	10	0	1	2	7
Medication management	0	0	0	10	0	0	1	9	0	0	2	8	0	0	1	9	0	0	2	8
Ask for help	0	0	1	9	0	0	1	9	0	0	2	8	0	0	0	10	0	0	2	8
Exercise	0	0	3	7	0	0	3	7	0	0	2	8	0	0	1	9	0	0	3	7
Relaxation training	0	0	3	7	0	0	3	7	0	0	1	9	0	0	3	7	0	0	0	10
Emotion diary	0	0	2	8	0	0	4	6	0	1	4	5	0	0	3	7	0	0	2	8
Change diet	0	2	3	5	0	0	8	2	0	1	5	4	0	1	4	5	0	0	3	7
**Anxiety**																				
Change cognition	0	0	1	9	0	0	0	10	0	1	0	9	0	0	0	10	0	0	0	10
Express emotions	0	0	0	10	0	0	0	10	0	0	0	10	0	0	0	10	0	1	0	9
Relaxation training	0	0	1	9	0	0	0	10	0	0	0	10	0	0	0	10	0	0	0	10
Professional help	0	0	0	10	0	0	0	10	0	0	1	9	0	0	1	9	0	0	0	10
Diet and exercise	0	0	3	7	0	0	2	8	0	0	2	8	0	0	3	7	0	0	2	8
**Sleep disorder**																				
Change cognition	0	0	2	8	0	0	1	9	0	0	0	10	0	0	0	10	0	0	0	10
Sleep habits	0	0	0	10	0	0	0	10	0	0	0	10	0	0	0	10	0	0	0	10
Sleep diary	0	0	1	9	0	0	2	8	0	1	3	6	0	0	0	10	0	0	1	9
Professional help	0	0	2	8	0	0	1	9	0	0	0	10	0	0	1	9	0	0	0	10
Complementary therapy	0	0	2	8	0	0	2	8	0	0	3	7	0	0	4	6	0	0	0	10
**Fever**																				

Professional help	0	0	0	10	0	0	0	10	0	0	0	10	0	0	0	10	0	0	0	10
Fever assessment	0	0	2	8	0	0	0	10	0	2	3	5	0	0	1	9	0	0	1	9
Medication management	0	0	1	9	0	0	0	10	0	0	2	8	0	0	0	10	0	0	0	10
Water consumption	0	0	4	6	0	0	4	6	0	0	0	6	0	0	0	6	0	0	0	10
Change cognition	0	0	2	8	0	0	3	7	0	0	3	7	0	0	1	9	0	3	3	4
**Diarrhea**																				
Professional help	0	0	0	10	0	0	1	9	0	0	1	9	0	0	0	10	0	0	0	10
Medication management	0	0	0	10	0	0	2	8	0	0	3	7	0	0	1	9	0	0	0	10
Diet adjustment	0	0	2	8	0	0	2	8	0	0	1	9	0	0	0	10	0	0	2	8
Skin care	0	0	2	8	0	0	0	10	0	0	1	9	0	0	0	10	0	0	0	10
Symptom diary	0	0	1	9	0	0	4	6	0	2	3	5	0	0	0	10	0	0	1	9
**Nausea or vomiting**																				
Professional help	0	0	0	10	0	0	0	10	0	0	0	10	0	0	0	10	0	0	0	10
Medication management	0	0	1	9	0	0	0	10	0	0	0	10	0	0	0	10	0	0	0	10
Diet adjustment	0	0	1	9	0	0	0	10	0	0	1	9	0	0	0	10	0	0	0	10
Complementary therapy	0	0	2	8	0	0	2	8	0	0	8	2	0	0	3	7	0	0	0	10
Symptom diary	0	0	0	10	0	0	1	9	0	1	3	6	0	0	0	10	0	0	2	8
**Shortness of breath**																				
Professional help	0	0	0	10	0	0	0	10	0	0	0	10	0	0	0	10	0	0	0	10
Medication management	0	0	0	10	0	0	0	10	0	0	0	10	0	0	0	10	0	0	0	10
Diet adjustment	0	0	1	9	0	0	1	9	0	0	0	10	0	0	0	10	0	0	0	10
Lifestyle	0	0	1	9	0	0	1	9	0	0	0	10	0	0	0	10	0	0	0	10
**Numbness/tingling of hands or feet**																				
Professional help	0	0	1	9	0	0	0	10	0	0	0	10	0	0	0	10	0	0	0	10

Self-management	0	0	1	9	0	0	0	10	0	0	0	10	0	0	0	10	0	0	0	10
Supplements	0	0	0	10	0	0	0	10	0	0	0	10	0	0	0	10	0	0	0	10
Relaxation training	0	0	0	10	0	0	0	10	0	0	0	10	0	0	0	10	0	0	0	10
**Headache**																				
Professional help	0	0	1	5	0	0	0	10	0	0	0	10	0	0	0	10	0	0	0	10
Medication management	0	0	1	5	0	0	0	10	0	0	0	10	0	0	0	10	0	0	0	10
Other measures	0	0	3	7	0	0	3	7	0	0	5	5	0	0	1	9	0	0	0	10
**Perianal neoplasms**																				
Professional help	0	0	0	10	0	0	0	10	0	0	0	10	0	0	0	10	0	0	0	10
Medication management	0	1	1	8	0	0	5	5	0	0	2	8	0	0	2	8	0	0	0	10
Lifestyle	0	0	0	6	0	0	0	10	0	0	0	10	0	0	0	10	0	0	0	10
Skin care	0	0	0	6	0	0	0	10	0	0	0	10	0	0	0	10	0	0	0	10
Diet adjustment	0	0	0	6	0	0	0	10	0	0	0	10	0	0	0	10	0	0	0	10
**Fat redistribution**																				
Know about the symptom	0	0	0	10	0	0	0	10	0	0	0	10	0	0	0	10	0	0	0	10
Professional help	0	0	0	10	0	0	1	9	0	0	3	7	0	0	3	7	0	0	0	10
Diet and exercise	0	0	0	10	0	0	0	10	0	0	3	7	0	0	0	10	0	0	0	10
Change habits	0	0	0	10	0	0	0	10	0	0	0	10	0	0	0	10	0	0	0	10
**Weight loss**																				
Professional help	0	0	2	8	0	0	0	10	0	0	0	10	0	0	0	10	0	0	0	10
Nutritional adjustment	0	0	2	8	0	0	0	10	0	0	0	10	0	0	0	10	0	0	0	10
Exercise	0	0	0	10	0	0	0	10	0	0	0	10	0	0	0	10	0	0	0	10
**Rash**																				
Professional help	0	0	0	10	0	0	0	10	0	0	0	10	0	0	0	10	0	0	0	10
Medication management	0	0	0	10	0	0	0	10	0	0	0	10	0	0	0	10	0	0	0	10

Diet	0	0	0	10	0	0	0	10	0	0	0	10	0	0	0	10	0	0	0	10
Symptomatic treatment	0	0	0	10	0	0	0	10	0	0	0	10	0	0	0	10	0	0	0	10
**Oral leukoplakia or ulcers**																				
Professional help	0	0	0	10	0	0	0	10	0	0	0	10	0	0	0	10	0	0	0	10
Oral hygiene	0	0	0	10	0	0	0	10	0	0	0	10	0	0	0	10	0	0	0	10
Diet adjustment	0	0	0	10	0	0	0	10	0	0	0	10	0	0	0	10	0	0	0	10
Pain management	0	0	3	7	0	0	1	9	0	0	0	10	0	0	0	10	0	0	0	10
**Muscle and joint pain**																				
Professional help	0	0	0	10	0	0	0	10	0	0	0	10	0	0	0	10	0	0	0	10
Medication management	0	0	1	9	0	0	2	8	0	0	0	10	0	0	0	10	0	0	0	10
Diet adjustment	0	0	0	10	0	0	0	10	0	0	0	10	0	0	0	10	0	0	0	10
Exercise	0	0	0	10	0	0	2	8	0	0	0	10	0	0	0	10	0	0	0	10
Change behavior	0	0	1	9	0	0	0	10	0	0	2	8	0	0	0	10	0	0	2	8
Supplements	0	0	4	6	0	0	3	7	0	0	5	5	0	0	4	6	0	0	0	10
**Memory loss**																				
Professional help	0	0	0	10	0	0	0	10	0	0	0	10	0	0	0	10	0	0	0	10
Medication management	0	0	1	9	0	0	0	10	0	0	0	10	0	0	0	10	0	0	0	10
Cognitive exercise	0	0	2	8	0	0	2	8	0	2	3	5	0	0	0	10	0	0	0	10
**Blurred vision**																				
Professional help	0	0	0	10	0	0	0	10	0	0	0	10	0	0	0	10	0	0	0	10
Medication management	0	0	0	10	0	0	0	10	0	0	0	10	0	0	0	10	0	0	0	10
Follow-up tracking	0	0	0	10	0	0	2	8	0	0	0	10	0	0	0	10	0	0	0	10
**Medication management**																				
Antiviral therapy	0	0	0	10	0	0	0	10	0	0	0	10	0	0	0	10	0	0	0	10
Medication introduction	0	0	1	9	0	0	0	10	0	0	0	10	0	0	0	10	0	0	0	10

Take medicine correctly	0	0	0	10	0	0	0	10	0	0	0	10	0	0	0	10	0	0	0	10
Emergency	0	0	3	7	0	0	2	8	0	0	3	7	0	0	0	10	0	0	1	9
**Complementary therapy**																				
Chinese traditional medicine	0	1	3	6	0	1	2	7	0	1	3	6	0	1	3	6	0	1	2	7
Vitamins and minerals	0	0	5	5	0	0	3	7	0	0	3	7	0	0	4	6	0	0	1	9
Antioxidants and amino acids	0	1	3	6	0	1	3	6	0	1	4	5	0	2	1	7	0	1	4	5
**Diet**																				
The importance of a reasonable diet	0	0	0	10	0	0	0	10	0	0	0	10	0	0	0	10	0	0	0	10
Safe diet	0	0	0	10	0	0	0	10	0	0	0	10	0	0	0	10	0	0	0	10
Balanced diet	0	0	0	10	0	0	0	10	0	0	1	9	0	0	0	10	0	0	0	10
Dishes recommendation	0	0	0	10	0	0	0	10	0	0	0	10	0	0	0	10	0	0	0	10
Diet and medication	0	0	0	10	0	0	1	9	0	0	1	9	0	0	2	8	0	0	1	9
Special diet	0	0	1	9	0	0	0	10	0	0	0	10	0	0	0	10	0	0	0	10
Rescourse and links	0	0	0	10	0	0	0	10	0	0	0	10	0	0	0	10	0	0	0	10
**Exercise**																				
The importance and principles of exercise	0	0	0	10	0	0	0	10	0	0	0	10	0	0	0	10	0	0	0	10
Healthy exercise	0	0	1	8	0	0	0	10	0	0	0	10	0	0	0	10	0	0	0	10
Exercise program	0	0	0	10	0	0	0	10	0	0	0	10	0	0	0	10	0	0	0	10
Precautions	0	0	0	10	0	0	0	10	0	0	0	10	0	0	0	10	0	0	0	10
Rescourse and links	0	0	0	10	0	0	0	10	0	0	0	10	0	0	0	10	0	0	0	10
**Relaxation technique**																				
Relaxation training	0	0	3	7	0	0	0	10	0	0	2	8	0	0	0	10	0	0	0	10

**Obtaining support**																				
Coping and adjustment	0	0	0	10	0	0	0	10	0	0	0	10	0	0	0	10	0	0	0	10
Professional support	0	0	0	10	0	0	0	10	0	0	0	10	0	0	0	10	0	0	0	10
Peer support	0	0	0	10	0	0	0	10	0	0	0	10	0	0	0	10	0	0	0	10
Resource and links	0	0	0	10	0	0	0	10	0	0	0	10	0	0	0	10	0	0	0	10
Questions and communications	0	0	0	10	0	0	0	10	0	0	0	10	0	0	0	10	0	0	0	10

Supplementary material 3

Table S3 Assessment tool and cutoff in the symptom management app

	Assessment tools	Cutoffs
**Symptoms**		
Anxiety	GAD-2: Over the last 2 weeks, how often have you been bothered by the following problem? (1) Feeling nervous, anxious or on edge; (2) Not being able to stop or control worrying. Linkert 4 (0-3).	(1) 0-3；(2) >3.
Depression	PHQ-2: Over the last 2 weeks, how often have you been bothered by the following problem? (1) Little interest or pleasure in doing things; (2) Feeling down, depressed or hopeless. Linkert 4 (0-3).	(1) 0-2; (2) ≥3.
Fatigue	(1) If a score of 0 means no fatigue at all, and a score of 10 means the worst fatigue you can imagine, how much do you rate your fatigue over the past 2 weeks? (2) How much does fatigue affect your daily life? (0 = not at all, 1 = a little, 2 = a moderate amount, 3 = very much, 4 = extremely)	(1) Item 1 scored 0; (2) Item 1 scored 1-3; (3) Item 1 scored 4-6; (4) Item 1 scored 7-10 or item 2 scored 3 or 4.
Headache	(1) If a score of 0 means no headache at all, and a score of 10 means the worst headache you can imagine, how much do you rate your headache over the past 2 weeks? (2) How much does affect affect your daily life? (0 = not at all, 1 = a little, 2 = a moderate amount, 3 = very much, 4 = extremely)	(1) Item 1 scored ≥1 or item 2 scored 1-4; (2) Other situations.
Fever	(1) What is your temperature? (2) How much does fever affect your daily life? (0 = not at all, 1 = a little, 2 = a moderate amount, 3 = very much, 4 = extremely)	(1) Fever (armpit temperature ≥ 37.2℃, or ear temperature, oral temperature, anal temperature ≥ 38℃) or item 2 scored 2-4. (2) Other situations.
Memory loss	(1) Do you think you're losing memory and forgetting important events more easily? (2) Is it difficult for you to concentrate? (3) Do people around you worry about your memory	(1) All items scored 0; (2) Other situations.

	or thinking ability? (4) How much does memory loss affect your daily life? Linkert 5 (0-4)	
Insomnia/can't sleep	(1) Do you need more than 30 min to fall asleep? What is the weekly frequency? (2) Do you easy to wake up during nights, and the awake time is more than 30 min? What is the weekly frequency? (3) Do you wake up early and feel tired after waking up? What is the weekly frequency? (4) How much does sleep problems affect your daily life? (0 = not at all, 1 = a little, 2 = a moderate amount, 3 = very much, 4 = extremely)	(1) Item 1 or item 2 or item 3 reported weekly frequency ≥3, or item 4 scored 1-4; (2) Other situations.
Blurred vision	(1) Do you have vision decline or blurred vision recently? (yes or no) (2) How much do you rate your blurred vision? (0 = not serious at all, 1 = a little serious, 2 = serious, 3 = quite serious, 4 = extremely serious) (3) How much does blurred vision affect your daily life? (0 = not at all, 1 = a little, 2 = a moderate amount, 3 = very much, 4 = extremely)	(1) Item 1 reported yes, or item 2 scored 1-4, or item 3 scored 1-4; (2) Other situations.
Rash, Itchy skin	(1) How much do you rate your rash? (0 = not serious at all, 1 = a little serious, 2 = serious, 3 = quite serious, 4 = extremely serious) (2) How much does rash affect your dialy life? (0 = not at all, 1 = a little, 2 = a moderate amount, 3 = very much, 4 = extremely) (3) If you have rash, do you have (a) no more symptoms, (b) itching or scaling, (c) blistering, extensive peeling, ulcers, severe itching (a=mild, b=moderate, c=severe) (4) Do you have fever or chest tightness?	(1) Item 1 scored 3-4, or item 2 scored 3-4, or item 3 reported c, or item 4 reported having chest tightness. (2) Other situations.
Mouth ulcers or white spots in mouth/thrush	(1) How much do you rate your mouth ulcers or white spots in mouth/thrush? (0 = not serious at all, 1 = a little serious, 2 = serious, 3 = quite serious, 4 = extremely serious) (2) How much does mouth ulcers or white spots in mouth/thrush affect your daily life? (0 = not at all, 1 = a little, 2 = a moderate amount, 3 = very much, 4 = extremely)	(1) Item 1 scored 1-4 or item 2 scored 1-4; (2) Other situations.

Muscle or joint pain	(1) If a score of 0 means no pain at all, and a score of 10 means the worst pain you can imagine, how much do you rate your muscle or joint pain over the past 2 weeks? (2) How much does muscle or joint pain affect affect your daily life? (0 = not at all, 1 = a little, 2 = a moderate amount, 3 = very much, 4 = extremely)	(1) Item 1 scored 0 and item 2 scored 0; (2) Item 1 scored 1-4; (3) Item 1 scored ≥ 6.
Numbness/tingling of hands or feet	(1) How much do you rate your numbness/tingling of hands or feet? (0 = not serious at all, 1 = a little serious, 2 = serious, 3 = quite serious, 4 = extremely serious) (2) How much does numbness/tingling of hands or feet affect your daily life? (0 = not at all, 1 = a little, 2 = a moderate amount, 3 = very much, 4 = extremely)	(1) Item 1 scored 1-4 or item 2 scored 1-4; (2) Other situations.
Weight loss	(1) What is your weight? What is your height? (BMI=weight/hight2, 0:BMI≥20, 1:BMI 18.5-20, 2:BMI≤18.5) (2) Do you lose weight in recent 3-6 months? How many kilogram have you lose in weight? (0:weight loss≤5%, 1:weight loss 5%-10%, 2:weight loss≥10%) (3)Undereating or fasting for more than 5 days. (0:yes, 2:no)	(1) 0; (2) 1; (3) ≥2.
Diarrhea	(1) What is the shape of your stool? (shaped stool, unshaped stool, watery stool, unshaped stool with slime, blood on stool surface, tarry stool) (2) Do you have abdominal pain or fever? (3) How much do you rate your diarrhea? (0 = not serious at all, 1 = a little serious, 2 = serious, 3 = quite serious, 4 = extremely serious)	(1) Item 1 reported blood on stool surface or tarry stool, or reported having abdominal pain or fever, or item 3 scored 3-4. (2) Other situations.
Nausea or vomiting	(1) How much do you rate your nausea or vomiting? (0 = not serious at all, 1 = a little serious, 2 = serious, 3 = quite serious, 4 = extremely serious) (2) How much does nausea or vomiting affect your daily life? (0 = not at all, 1 = a little, 2 = a moderate amount, 3 = very much, 4 = extremely)	(1) Item 1 scored 1-4 or item 2 scored 1-4; (2) Other situations.
Fat accumulation in the abdomen, back and neck or atrophy of the face and limbs	(1) Do your legs and/or arms become thinner or fat shrink? (2) Does your tummy become bigger or neck become thicker? (3) How much do you rate your fat redistribution? (0 = not serious at all, 1 = a little serious, 2 = serious, 3 = quite serious, 4 = extremely serious) (4) How much does fat redistribution affect your daily life? (0 = not at all, 1 = a little, 2 = a moderate amount, 3 = very much, 4 = extremely)	(1) Item 1 reported yes, or item 2 reported yes, or item 3 scored 1-4, or item 4 scored 1-4; (2) Other situations.

Shortness of breath	(1) When do you feel shortness of breath? (brisk walking: level I, normal speed walking: level II, stop normal speed walking due to shortness of breath: level III, slight activity: level IV) (2) How much does shortness of breath affect your daily life? (0 = not at all, 1 = a little, 2 = a moderate amount, 3 = very much, 4 = extremely)	(1) Item 1 reported Level II, III or IV, or item 2 scored 1-4; (2) Other situations.
Perianal neoplasms	(1) How much do you rate your perianal neoplasms? (0 = not serious at all, 1 = a little serious, 2 = serious, 3 = quite serious, 4 = extremely serious) (2) How much does perianal neoplasms affect your daily life? (0 = not at all, 1 = a little, 2 = a moderate amount, 3 = very much, 4 = extremely)	(1) Item 1 scored 1-4 or item 2 scored 1-4; (2) Other situations.
